# Biodegradable PTLGA Terpolymers versus Collagen Implants Used as an Adjuvant in Trabeculectomy in Rabbit Eye

**DOI:** 10.1155/2015/737198

**Published:** 2015-11-30

**Authors:** Weiran Niu, Guanglin Shen, Yuanzhi Yuan, Xiaoping Ma, Suming Li, Jingzhao Wang, Zhongyong Fan, Lan Liao

**Affiliations:** ^1^Department of Ophthalmology, Zhongshan Hospital of Fudan University, Shanghai 200032, China; ^2^Institute de High Performance Polymers, Qingdao University of Science and Technology, Qingdao 266042, China; ^3^Department of Materials Science, Fudan University, Shanghai 200433, China

## Abstract

*Purpose*. To evaluate the effectiveness and safety of three biodegradable terpolymers prepared from L-lactide, trimethylene carbonate, and glycolide (PTLGA) as an aid for trabeculectomy compared with the Ologen (OLO).* Methods*. Trabeculectomy was carried out on rabbits with implantation made from OLO or three PTLGA terpolymers. Intraocular pressure (IOP) was recorded 1, 2, 3, and 6 months postoperatively and bleb evaluations were performed using ultrasound biomicroscopy (UBM) 3 months after surgery, optical coherence tomography (OCT) every month, and transmission electron microscopy (TEM) six months after surgery followed by histological examination 1, 2, 3, and 6 months postoperatively.* Result*. IOP was significantly reduced in all groups after surgery. There were no significant differences in the IOL between groups at any time after implantation. There was no significant difference between the groups examined by OCT, UBM, and TEM. Exposure of the implant was observed in one eye from the OLO group and one eye in the P1. Subconjunctiva hyperblastosis was observed in one eye from group P3 and two eyes from the OLO group.* Conclusions*. Subconjunctival implantation of filtering devices made from PTLGA may present a safe and effective additional surgical tool for the treatment of filtering surgery. Fewer complications were observed in the group with P2 implants compared to other groups.

## 1. Introduction

Since trabeculectomy was introduced in 1968, it has been the most common surgery to treat glaucoma [1]. Because scarring is still the major threat to the long-term success of glaucoma trabeculectomy surgery, mitomycin C (MMC) has been widely used as an antifibrotic agent to inhibit fibroblast proliferation and to maintain an ideal postoperative intraocular pressure (IOP) for more than two decades [2]. It has been proved that MMC raised the success rate of many types of glaucoma filtration surgery [3–5], and the use of MMC as a therapeutic agent has been well established as an effective clinical practice at achieving low final intraocular pressures (IOPs). However, severe complications such as leakage, infection, hypotony, and endophthalmitis with complete loss of vision may occur in surgeries with MMC, and surgery still fails in some individuals unfortunately [6, 7]. Less toxic antifibrotic agents or better therapeutic methods are needed.

Because of that, many promising new agents and implants to inhibit scar formation with fewer adverse effects have been evaluated in recent researches. Biodegradable implants such as Ologen (OLO), a porous collagen-glycosaminoglycan matrix, could potentially take the place of MMC to prevent the adhesion of the conjunctiva and sclera [8, 9] and the collapse of the subconjunctival space after trabeculectomy which leads to collagen deposition and microcyst formation after penetrating antiglaucomatous surgery. Use of OLO decreased early postoperative scarring and could be used to repair postoperative bleb leaks [10, 11]. Adverse effects of OLO included translocation or exposure of the implant or erosion of the conjunctiva.

Biodegradable polymers such as poly(L-lactide) (PLLA), polyglycolide (PGA), poly(1,3-trimethylene carbonate) (PTMC), and their copolymers have attracted great attention for biomedical applications such as surgical implants, drug carrier, and tissue engineering scaffold because of their outstanding biocompatibility and biodegradability. In previous studies, terpolymers prepared from L-lactide, glycolide, and 1,3-trimethylene carbonate (PTLGA) have been successfully used as intravascular stent [12].

The goal of this study was to evaluate the efficacy and safety of PTLGA terpolymer in keeping the postoperative space between the conjunctiva and sclera functional and thus to maintain the filtering bleb active after trabeculectomy in rabbit eyes.

## 2. Materials and Methods

All experiments were carried out on the OD eye under a surgical microscope and general anesthesia. Rabbits weighing between 2.5 and 3.0 kg were anesthetized by intramuscular injection of ketamine hydrochloride (10 mg/kg) and xylazine (20 mg/kg). Topical anesthesia (0.5% proparacaine hydrochloride, Alcaine, Alcon-Couvreur, Belgium) was applied to the eyes. All rabbits were treated in accordance with the ARVO Statement on the Use of Animals in Ophthalmic and Vision Research, and the experimental protocol was approved by the Animal Care and Use Committee of Zhongshan Hospital, Fudan University.

### 2.1. Trabeculectomy in Rabbit Eyes

Four groups of eight female rabbits underwent trabeculectomy (32 animals in total). A fornix-based conjunctival incision was made and a 3 × 3 mm scleral flap was created. Trabeculectomy was performed at the scleral spur, followed by iridectomy. A 5 × 6 mm implant sheet was placed on the scleral flap, the implant inserted over the flap. The scleral flap and conjunctival wound were sutured with 10-0 nylon. The implants included OLO and 3 different kinds of PTLGA terpolymers, P1, P2, and P3, in the study groups (see Section 2.2 below). No antifibrotic agent such as MMC was applied. Filtering bleb formation and inflammation of the anterior chamber were observed with slit-lamp microscopy by masked observers.

### 2.2. Materials

**Figure 1:**
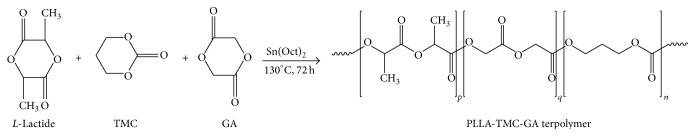
Synthesis route of PTLGA terpolymers. By permission from Shen et al. [12].

PTLGA terpolymers [13] (Figure 1) were generously prepared [12] and provided by Dr. Suming Li and Dr. Zhongyong Fan. The implant has a shape of film and a thickness of 1 mm:

 P1, PLLA : GA : TMC (95.8 : 5.4 : 4.2, mole : mole). Number average molecular weight is 232 KDa.
 P2, PLLA : GA : TMC (60 : 13.2 : 22.8, mole : mole). Number average molecular weight is 45 KDa.
 P3, PLLA : GA : TMC (2 : 1.19 : 0.8, mole : mole). Number average molecular weight is 28 KDa.

OLO (Ologen; Aeon Astron Group B.V. Leiden, the Netherlands). The implant size was 10 mm (*W*) × 10 mm (*L*) × 2 mm (*H*) (number 870051).

All implants were cut into 5 mm (*W*) × 6 mm (*L*) in the study.

### 2.3. Examining Methods

#### 2.3.1. Intraocular Pressure (IOP) Measurements

After topical anesthesia of 0.5% proparacaine hydrochloride Alcaine (Alcon-Couvreur, Belgium), IOP was measured using a Goldman applanation tonometry at baseline and twice a month for 1, 2, 3, and 6 months after surgery in all groups. IOP measurements were made by a blinded investigator. An average of three tonometer readings, with a maximum 5% standard deviation (SD), was recorded per eye. The IOP of all the experimental rabbits was measured at approximately 3 p.m.

#### 2.3.2. Hematoxylin-Eosin Staining

Rabbits were killed by excess ketamine (35 mg/kg) and xylazine (5 mg/kg) on months 1, 2, 3, and 6 after implantation. Eyes were quickly removed and fixed in 4% formaldehyde. The conjunctiva and implant with the underlying scleral bed were dissected, dehydrated, and embedded in paraffin. Sections were cut by a microtome at 7 micrometers and stained with hematoxylin and eosin (H&E) for general histologic observation.

#### 2.3.3. Optical Coherence Tomography (OCT)

The subconjunctival fluid space was classified as none, single small, multiple small, or large and scored as 0, 1, 2, and 3, respectively, using a Zeiss Visante OCT (Model 1000, Carl Zeiss Meditec Inc., Dublin, CA). The upper lid was gently lifted by the operator to maximize bleb exposure as best as possible without pressing on the globe. Several radial and transverse sections were assessed.

#### 2.3.4. Ultrasound Biomicroscopy (UBM)

**Figure 2:**
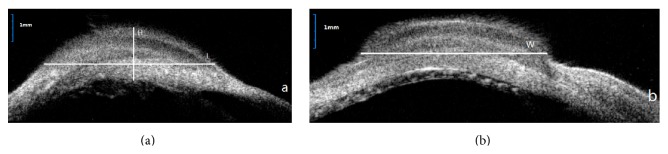
Imaging of UBM examination. (a) Horizontal section of the filtering bleb. (b) Vertical section of the filtering bleb.

UBM (SW-3200, SUOER, Tianjin, China) was used to evaluate the height of the postoperative filtering blebs, and the maximum height of the filtering bleb including the eyeball wall in each scan was manually measured and automatically calculated with the device's software. The vertical line was drawn through the maximum height point of the filtering bleb at the corneal limbus, whereas a horizontal line through the same point was used as a base (Figure 2); the bleb area and height within this section of the bleb were used to estimate the volume of the bleb area and analyzed.

#### 2.3.5. Transmission Electron Microscope Examination

Rabbits were euthanized six months after surgery. Eyes were quickly removed and fixed in 4% formaldehyde. Sections of conjunctiva and implant with the underlying scleral bed were cut to a thickness of 1 *μ*m stained with uranyl acetate, followed by lead citrate. Slices were observed using an electron microscope (JEOL 1200, JEOL Co., Japan).

### 2.4. Statistical Analysis

Statistical analyses were performed using SPSS statistical software (Windows version 20.0; SPSS Inc., Chicago, IL, USA). One-way analyses of variance (One-way ANOVA) and Fisher's exact test were used to compare between the groups. In comparison of pre/postoperative results, paired Student's* t*-test was used. Complete resorption time was compared using the log rank test and survival curves (Kaplan-Meier curves). The results are expressed as mean ± standard deviation, and *P* < 0.05 was deemed to be statistically significant.

## 3. Results

### 3.1. IOP Measurements

No animals were excluded from this study since there were no intraoperative complications. One month after surgery, postoperative mean intraocular pressure (IOP) was significantly lower (*P* < 0.0001) in all groups. At one, three, and six months after surgery, the mean IOP was not statistically (*P* > 0.05) different between groups (Table 1).

### 3.2. Morphology Observation

**Figure 3:**
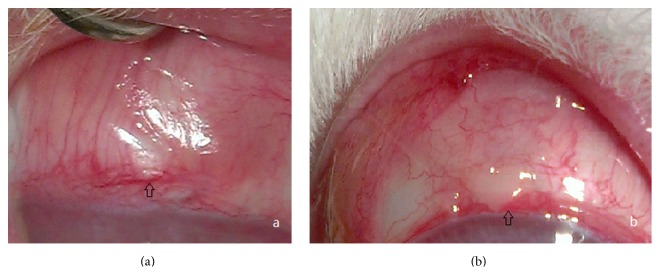
Exposure of implant happened in 2 eyes; both of them were repositioned by conjunctival suture and recovered well. (a) Exposure of Ologen in control group. (b) Exposure of P1 in Group 1.

Only mild conjunctival hyperemia and edema were observed three to six days postoperatively and disappeared about two weeks later. No occurrence of keratitis, uveitis, or endophthalmitis was observed. During the postoperative follow-up observations, we did not detect allergy or translocation of the implants. Exposure of the implant was observed in one eye in the control group in the second week and group 1 in forth week. The implants were repositioned by conjunctival suture (Figure 3).  Table 2 provided an overview of the recorded side effects between the OLO group and PTLGA terpolymer groups. Using the Kruskal-Wallis test, the frequency of postoperative complication did not significantly differ between the groups.

**Figure 4:**
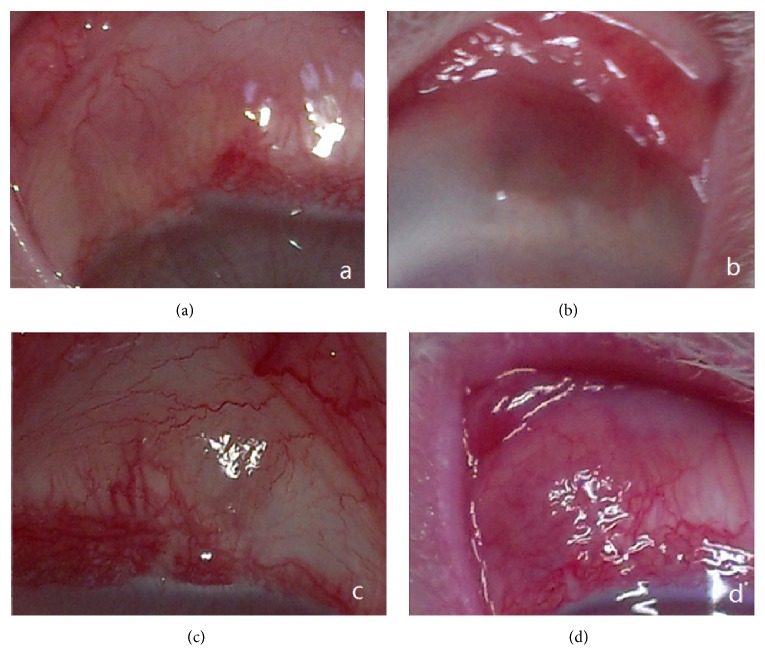
Morphology observation of the filtering bleb 3 months after surgery. (a) Control group. (b) Group 1. Implant under the conjunctiva still can be seen. (c) Group 2. (d) Group 3.

One month after surgery, the filtering bleb was obviously visible in all eyes. Three months later, the filtering blebs were observed flatter in eyes of the OLO group and groups P2 and P3 (Figure 4). Six months after surgery, except for group P1, it was quite difficult to observe the filtering blebs.

### 3.3. Histologic Evaluation

**Figure 5:**
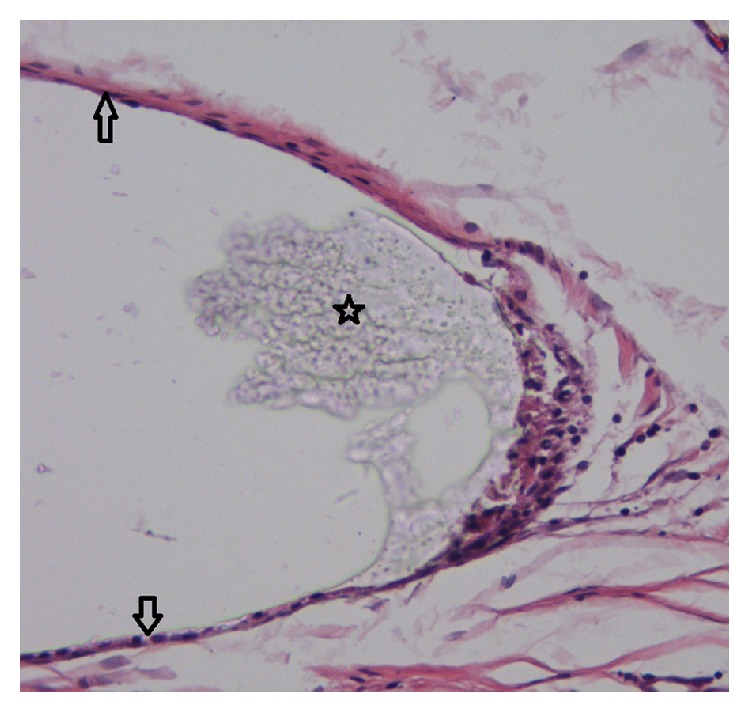
Weak fibrous capsule around the biomaterials marked by arrows.

**Figure 6:**
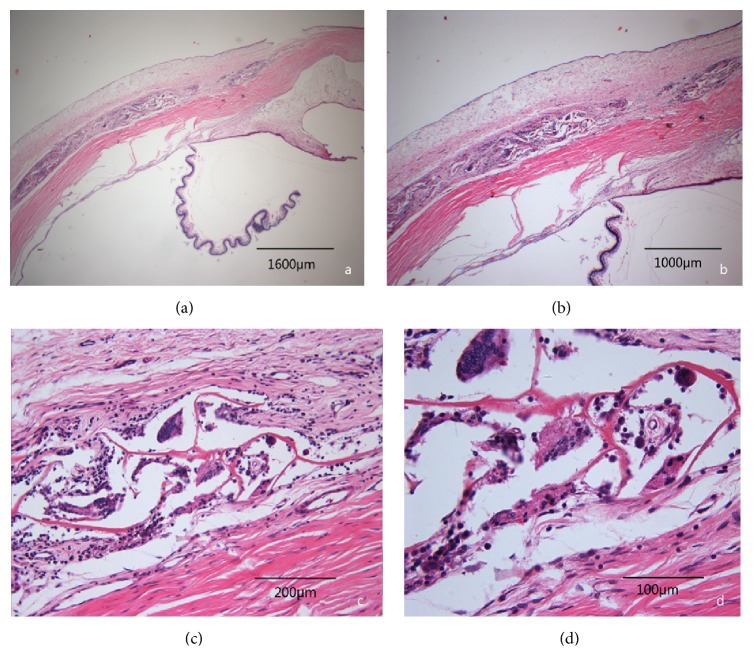
Subconjunctiva hyperblastosis composed of fibroblasts, small vessels, and inflammatory cells had been observed in the control group; the biomaterials (Ologen) had already degraded. Hematoxylin-Eosin staining, (a) ×25. (b) ×40. (c) ×200. (d) ×400.

With hematoxylin-eosin staining we observed that all the biomaterials were found in the samples isolated at three months, except for P1 which had been observed at 3 months after surgery and undergone complete degradation six months after implantation. A weak fibrous capsule formation was observed around all of the biomaterials especially after one month after implantation (Figure 5). Three months after surgery, the fibrous capsule was difficult to observe in all eyes of group P3 and 3 eyes of the OLO group. Subconjunctiva hyperblastosis composed of fibroblasts, small vessels, and inflammatory cells was observed in one eye of group P3 and 2 eyes of the OLO group even after the biomaterials were biodegraded (Figure 6).

### 3.4. Morphology Examination

#### 3.4.1. Optical Coherence Tomography (OCT)

**Figure 7:**
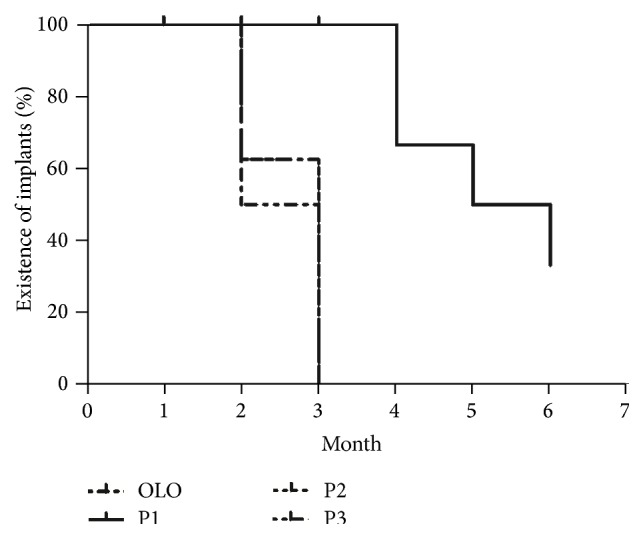
The Kaplan-Meier chart of complete resorption time between the Ologen group and PTLGA terpolymer groups. There were no significant differences of the curves between those groups (log rank test, *P* = 0.051).

**Figure 8:**
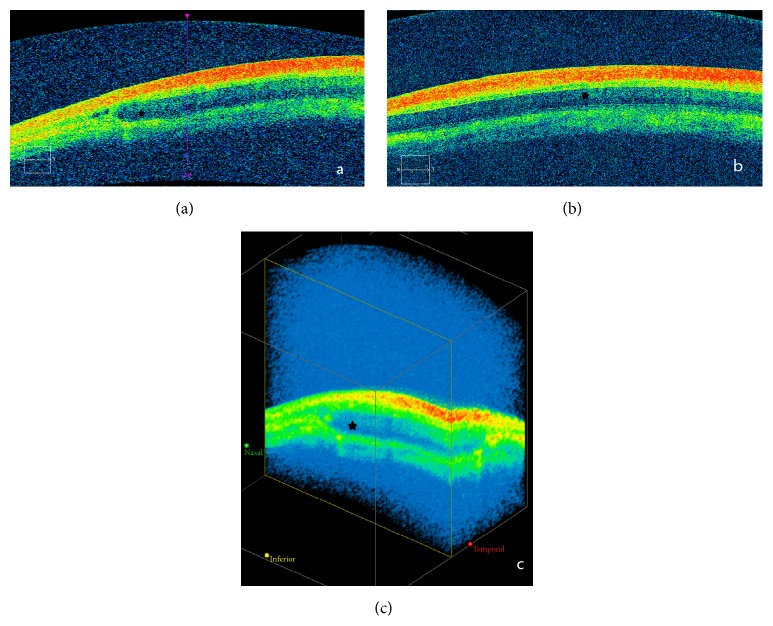
Imaging of OCT examination. (a) Horizontal section of the subconjunctival fluid space. (b) Three-dimensional reconstruction of the subconjunctival fluid space. (c) Vertical section of the subconjunctival fluid space.

Gradual resorption of the implant and subconjunctival fluid spaces was observed. The complete resorption time was 2 months in 10 eyes and 3 months in 14 eyes. The complete degradation time of group P1 was longer than others but not significantly different, 4 months in 2 eyes, 5 months in 1 eye, and 6 months in 1 eye; there were still implants left in 2 eyes when rabbits were executed. There were no significant differences of the Kaplan-Meier chart curves between the Ologen group and PTLGA terpolymer groups (log rank test, *P* = 0.051) (Figure 7). Three months after surgery, the OCT scores were not significantly (*F* = 0.77, *P* = 0.53) different between groups: 2 ± 1, 2.5 ± 0.6, 1.8 ± 0.5, and 2.0 ± 0.8 for the groups OLO, P1, P2, and P3, respectively. Because of its low depth of penetration, OCT could not be used to assess the route under the scleral flap in some cases, but it is easy to examine if there are implantations left (Figure 8).

#### 3.4.2. Ultrasound Biomicroscopy (UBM)

There were no significant (*F* = 2, *P* = 0.18) differences in mean bleb volume at three months: 26 ± 7 mm^3^, 21 ± 7 mm^3^, 33 ± 3 mm^3^, and 30 ± 11 mm^3^, for groups OLO, P1, P2, and P3, respectively.

### 3.5. Transmission Electron Microscope

**Figure 9:**
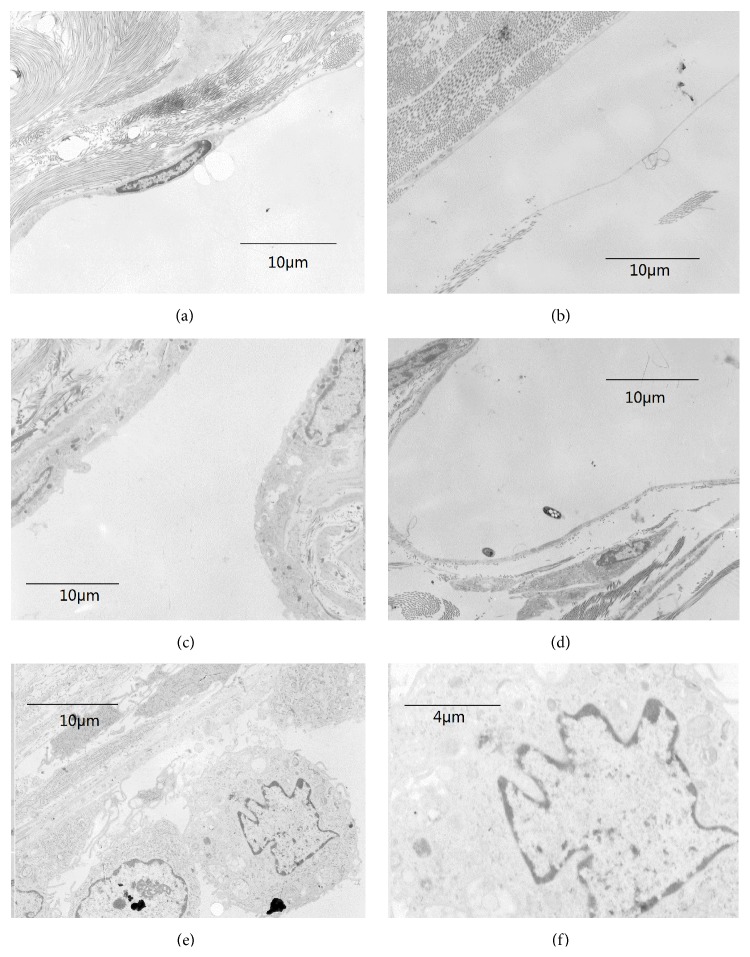
Transmission electron microscope. A weak fibrous capsule formation marked by arrows was observed around all of the 4 biomaterials: (a) control group, ×2500; (b) group 1, ×2500; (c) group 2, ×2500; (d) group 3, ×2500. And small amounts of macrophages marked by arrows had been observed nearby ((e) ×2500, (f) ×5000).

A weak fibrous capsule formation was observed around all of the four biomaterials, and small amounts of macrophages were observed nearby (Figure 9).

## 4. Discussion

As stated in the introduction, trabeculectomy is the most common antiglaucoma surgical procedure. Although both of MMC and OLO had a good record of higher achievement ratio in the surgery [14, 15], because of some adverse effects of them, investigators have developed new materials to take their place. One intravascular stent material, PTLGA terpolymer, which has a good record of safety and histocompatibility [12], was tested as a biodegradable implant compared to OLO.

Published studies of our department had proved that there are reasons why trabeculectomy with Ologen has been a safe and effective procedure in patients with glaucoma even during a five-year follow-up. First, the structure of Ologen contained thousands of microscopic pores and can induce fibroblast growth, leading to a well organized and healthy healing process. Second, with a thickness of 2 mm and placed directly over the scleral flap and under the subconjunctival space, Ologen could provide space with a dynamic and physiological aqueous reservoir system. Subsequently, Ologen is biodegraded by the body within 90~180 days from its implantation which offered enough time to create a mature bleb structure [16]. In this study, we tested the PTLGA terpolymers, which have a similar structure and a similar degradation time to Ologen and have a thinner thickness of 1 mm, so, theoretically, these implants can get the similar outcome as Ologen in improving the surgical success of trabeculectomy without the adjunctive use of antifibrotic agents.

In this study, biodegradable implants made of PTLGA terpolymers were as safe as those made of OLO. Implants of PTLGA terpolymers were as effective as those made of OLO in achieving a low target IOP level. It was encouraging to confirm that P1 and P2 maintained the size of filtering blebs, even though there might be no strong correlation between IOP and bleb height [17]. The differences of the physical and chemical properties between the PTLGA terpolymers were due to the different percentages of components in the PTLGA terpolymer. The results of examinations and complications in the study included that the efficacy and safety of P2 made it the most suitable implant among the three kinds of PTLGA terpolymers.

Exposure of the implant was one of the complications we found. Exposure in the OLO group may have been due to the thickness of implant. Exposure of implant made from P1 may be due to the hardness of P1 with sharp edges, cusp angles, and a long biodegradation procedure. There were no significant differences of complete resorption time between the groups (we did not take the two rabbits killed before complete resorption of implants into account as perfect biodegraded); we presume that differences would be found and the longest complete resorption time would be seen in the P1 group, if our study has a bigger sample and a longer follow-up time. And we also presume that if the P1 implants were roundish shaped and with a blunt edge, the exposure of P1 would be avoidable.

Subconjunctiva hyperblastosis was a unique complication observed in our study which may be due to the response of the conjunctiva to the long-term stimulus from the more acid decomposition product of P3, because P3 has the highest percentage of GA. Subconjunctiva hyperblastosis has been observed in both P3 and OLO groups; however, it is arduous to find such reports in published studies; there may be some reasons behind this: in the first place, OLO has a fair histocompatibility itself; the low occurrence of subconjunctiva hyperblastosis could make it difficult to be observed; then, the subconjunctiva hyperblastosis did not raise the IOP so obviously or so frequently after the operation; third, there might be an absorption of it; the hyperblastosis could only be observed in a short time; the last, it might be hard to be observed by other testing methods. Although the subconjunctiva hyperblastosis in our research did not cause the significant difference of success rate between the groups, we cannot help but conceive the idea that when the subconjunctiva hyperblastosis grows large enough to block the aqueous humor outflow channel, the surgery might end in failure. Longer time follow-up observation was needed to confirm whether there will be a regression or a growth of the subconjunctiva hyperblastosis later, to find what stimulated the development of subconjunctiva hyperblastosis and what strategy is needed for avoiding it or controlling it.

There was a good control of IOP after surgery and no significantly different frequency of complications in all the 4 groups. In particular, complications such as exposure were not observed for implants made of P2, as supposed, due to the thin and soft characteristic of it, and there was no subconjunctiva hyperblastosis observed in the P2 group either, which has a chance to block the aqueous humor outflow of the artificial channel built in the trabeculectomy and cause the IOP to rise rapidly. All these results suggested that P2 may be an ideal material as a new choice of antifibrotic agents. Due to the low incidence of complications and small sample size of our study, more studies are needed to confirm this idea.

OCT measurements were more reproducible and easier to perform than those of UBM. Because of its low depth of penetration, although OCT could not be used to assess the route under the scleral flap in some cases, it is competent enough to observe the residue of implants under the conjunctiva. Detailed anatomic assessment of bleb morphology was made using UBM and OCT. Several investigators have established that UBM and OCT can be used to identify morphologic changes in blebs related to wound healing and to identify parameters for the functional prognosis of the filter blebs [18–22]. The complementary use of OCT and UBM made them irreplaceable.

It is widely accepted that OLO keeps an outstanding postoperative IOP; according to the results of our study, all the three kinds of PTLGA terpolymers played a similar role in the control of IOP after antiglaucoma surgical procedure. Results also implied that there were fewer adverse events in PTLGA terpolymer groups happening than that in the OLO group. Since P2 manifested the fewest complications among all the agents, consequently it has been considered as the most promising adjuvant in trabeculectomy.

Our study had several limitations. Our sample size was relatively small, and the study reported short-term outcomes.

## 5. Conclusions

In conclusion, OCT and UBM were useful tools to measure filtering blebs. Subconjunctival implants made from PTLGA may present a safe and effective additional surgical tool for the maintenance of filtering blebs in antiglaucoma surgery. Fewer complications were observed in the group with P2 implants. Longer-term observations of bigger sample size studies are needed to fully evaluate this new PTLGA terpolymer.

## Figures and Tables

**Table 1:** Comparison of IOP measurements between the Ologen group and PTLGA terpolymer groups.

Time points	*n*	OLO (mean ± SD)	P1 (mean ± SD)	P2 (mean ± SD)	P3 (mean ± SD)
Preoperative	8	15.5 ± 1.2	15.3 ± 1.3	15.4 ± 1.3	15.4 ± 1.1^*※∗*^
1 month after surgery	8	9.6 ± 1.1	9.3 ± 1.6	9.6 ± 1.5	9.7 ± 1.1^*※∗*^
2 months after surgery	6	13.5 ± 1.2	13.3 ± 2.0	13.6 ± 0.9	13.7 ± 1.0^*∗*^
3 months after surgery	4	15.1 ± 1.1	14.5 ± 1.9	15.1 ± 1.3	15.2 ± 1.2
6 months after surgery	2	16.3 ± 0.6	13.9 ± 0.5	16.2 ± 0.1	15.9 ± 1.5

^*※*^ *P* = 0.000, paired-sample *t*-test.

^*∗*^ *P* < 0.05, paired-sample *t*-test.

Data are presented as mean ± standard deviation.

**Table 2:** Comparison of complications between the Ologen group and PTLGA terpolymer groups.

Complications	OLO number (%)	P1 number (%)	P2 number (%)	P3 number (%)	*P*
Mild conjunctival hyperemia	7 (87.5%)	8 (100%)	7 (87.5%)	7 (87.5%)	0.057
Mild conjunctival edema	2 (25%)	5 (62.5%)	3 (37.5%)	1 (12.5%)	1.000
Mild hyphema	0	0	0	0	—
Hypotony	0	0	0	0	—
Shallow anterior chamber	0	0	0	1 (12.5%)	0.057
Avascular cystic bleb	0	0	0	0	—
Cataract formation	0	0	0	0	—
Keratitis	0	0	0	0	—
Uveitis	0	0	0	0	—
Endophthalmitis	0	0	0	0	—
Choroidal detachment	0	0	0	0	—
Bleb leakage	1 (12.5%)	1 (12.5%)	0	1 (12.5%)	0.057
Implant exposure	1 (12.5%)	1 (12.5%)	0	0	0.086

Fisher's exact test.
